# *Listeria monocytogenes* in Export-approved Beef from Mato Grosso, Brazil: Prevalence, Molecular Characterization and Resistance to Antibiotics and Disinfectants

**DOI:** 10.3390/microorganisms8010018

**Published:** 2019-12-20

**Authors:** Larrayane A.C. Teixeira, Fernanda T. Carvalho, Deyse C. Vallim, Rodrigo C.L. Pereira, Adelino Cunha Neto, Bruno S. Vieira, Ricardo C.T. Carvalho, Eduardo E.S. Figueiredo

**Affiliations:** 1 College of Nutrition, Federal University of Mato Grosso, 78060-900 Cuiabá, MT, Brazilfefetavares_carvalho@hotmail.com (F.T.C.); adeneto40@gmail.com (A.C.N.);; 2 Laboratory of Bacterial Zoonoses, Oswaldo Cruz Institute, Oswaldo Cruz Foundation, 21040-360 Rio de Janeiro, RJ, Brazil; vallim@ioc.fiocruz.br (D.C.V.);; 3 College of Animal Science, Federal Institute of Education, Science and Technology of Mato Grosso, 78580-000 Alta Floresta, MT, Brazil

**Keywords:** antimicrobial resistance, beef, food safety, *Listeria monocytogenes*, PFGE, sodium hypochlorite

## Abstract

The Brazilian state of Mato Grosso is the largest producer and exporter of beef in the country, but few studies of relevance have been conducted to evaluate the microbiological safety of its products. This study aimed to estimate the prevalence of *Listeria monocytogenes* (LM) in export-approved beef from Mato Grosso and to characterize the isolates in terms of molecular properties and antimicrobial resistance. From a total of 50 samples analyzed, *Listeria* sp. was isolated in 18 (36% prevalence). *Listeria monocytogenes* was confirmed in 6 (12% prevalence). Among the serotype groups assessed by multiplex PCR, serotype 4 (4b, 4d or 4e) was the most prevalent. Although antibiotic resistance was not an issue, two strains isolated from different plants showed high resistance to sodium hypochlorite. Overall, this scenario causes concern because it puts at risk not only the Brazilian customer, but also the population of countries that import beef from Mato Grosso.

## 1. Introduction

*Listeria monocytogenes* (LM) is the causative agent of listeriosis, a foodborne disease that affects mainly at-risk populations such as the elderly, pregnant woman, newborns and immunocompromised individuals [1]. Due to the high susceptibility of these population groups and the potential of LM to generate severe bacteremia and systemic infections, mortality associated with the invasive form of listeriosis is generally high, reaching 25–30% of the patients [2].

Animal-source food has been implicated in many of the clinical cases of listeriosis in humans. The presence of LM has already been described in milk, cheese, meat, fish and sausage [3,4,5]. A characteristic trait of LM that increases its odds to persist in food processing environments even after sequential cleaning and disinfection measures is the ability to produce biofilms, which protects the bacterium from the action of disinfectants. In this situation, not only the risk of food contamination increases, but the chances of resistant strains to arise are greater [6,7].

Based on that, description of LM resistance to disinfectants—which was previously considered a rare event—has been increasing in the literature [8]. The same situation is happening with LM resistance to antibiotics [9,10]. Whether this last finding is the consequence of extensive usage of antibiotics in human medicine, in veterinary medicine, or cross-resistance to authorized molecules used as growth promoters in animal production is still an open discussion [11,12]. In any case, this lack of clarity only reinforces the need for regular inspection of LM food sources and study of isolates’ profile in order to establish proper measures to reduce the risk of food contamination and to improve the effectiveness of treatments by health services.

According to the Brazilian Institute of Geography and Statistics, the state of Mato Grosso holds the largest cattle herd and leads the beef-export activity in the country, accounting for 16.6% of the total volume of beef exported by Brazil in 2016 [13]. Despite the multiple international regulations followed by export-authorized plants, Brazilian internal regulations only require the control of LM in ready-to-eat products [14,15]. Not surprisingly, recent surveys in Brazil [16] demonstrated that the prevalence of LM in Brazilian packaged beef is very similar to that of 16% estimated for the global prevalence of LM in fresh beef [17]. This scenario may put at risk not only the Brazilian population, but also the consumers from other countries that import fresh beef from Brazil, a group currently represented by 74 countries including China, the Netherlands, Italy and Turkey.

The objectives of this study were to estimate the prevalence of *Listeria monocytogenes* in export-approved beef from Mato Grosso, Brazil, to identify the circulating serotypes and lineages, to compare the genotypic characteristics of isolates and to characterize their profile of resistance to antibiotics and disinfectants.

## 2. Materials and Methods

### 2.1. General Procedures

During a six-month evaluation period, 50 beef samples from 13 different export-authorized processing plants in the state of Mato Grosso, Brazil were obtained from the regular material used in the pathogen monitoring program of the official inspection service. Samples (flank steak, 1–2 kg each) were aseptically collected immediately after packaging, put into sterile plastic bags and kept on ice for approximately 12 h before laboratory processing.

### 2.2. Listeria sp. Isolation and Species Confirmation

To investigate the occurrence of LM in the collected material, all samples were subjected to traditional and widely recommended procedures of isolation and identification of the pathogen [18], using half Fraser and Fraser broths (Difco, France) as selective enrichment media. First, aliquots of 25 g of each sample were included in 225 mL of half Fraser broth and incubated at 37 °C for 24 h. After this period, 0.1 mL of this primary enrichment broth was transferred into tubes containing 10 mL of Fraser broth and incubated at 37 °C for 24 h. At the end of this protocol, tubes showing dark color were considered presumptively positive for *Listeria* sp. Confirmation was obtained after differential plating (35 °C for 48 h) on Modified Oxford Agar (Oxoid, Basingstoke, UK) and *Listeria* Agar (Biocen, Campinas, Brazil). Typical black colonies, small in the size and surrounded by a dark halo of aesculin hydrolysis, were considered as *Listeria* sp.

From each plate with characteristic growth, three colonies were isolated and further treated for Gram’s staining and motility test. In addition, all the isolates were submitted to the biochemical proofs of catalase, hemolysis on 5% Sheep Blood Agar (SBA), and xylose/rhamnose fermentation. To enhance the hemolytic activity of positive strains and resolve undetermined results, traditional CAMP test was performed for all isolates [19]. Gram positive colonies with the presence of umbrella-like motility, positive results for catalase, hemolysis (SBA or CAMP test) and rhamnose fermentation; negative results for xylose fermentation were considered as LM.

Isolates were stocked in Brain Heart Infusion (BHI) broth + 0.5% Yeast Extract + 20% Glycerol at −80 °C for LM confirmatory analysis using real-time PCR.

### 2.3. Real-time PCR Confirmatory Assay

Bacterial DNA was extracted from all isolates by thermal lysis and quantified by fluorometry using a Qubit device (Invitrogen, Singapore). Molecular confirmation of LM was performed in triplicate by real-time PCR, following the procedures originally described by Traunsek et al. [20] and modified by Moura et al. [21].

In short, one fragment of the *hlyA* gene was amplified using primers and species-specific probe. Real-time PCR was optimized with 2× universal PCR master mix, 600 nM of each primer, 200 nM of the probe and 40 ng of bacterial DNA. Amplification conditions were set to 10 min at 95 °C, followed by 45 cycles of 15 s at 95 °C and 1 min at 60 °C. Reactions were carried out in a 7500 Real Time PCR machine (Applied Biosystems, Waltham, MA, USA). The assay was controlled using DNA from LM (ATCC 7644), exogenous internal positive control reagents and DNase/RNase-free distilled water (Invitrogen, São Paulo, Brazil).

### 2.4. Molecular Serotyping

Multiplex PCR was carried out to assess both the serotype and lineage of the confirmed LM isolates. The protocol adopted here was originally proposed by Doumith et al. [22] and uses the amplification profile of the genes *lmo0737, lmo1118, ORF2819* and *ORF2110* to separate LM into four groups: i) serotypes 1/2a or 3a, ii) serotypes 1/2c or 3c, iii) serotypes 1/2b, 3b or 7, iv) serotypes 4b, 4d or 4e.

For that, bacterial DNA was extracted using a DNeasy Blood & Tissue kit (Qiagen, Hilden, Germany), following the manufacturer’s recommendations. The PCR assay was performed in a final volume of 50 µL: 1× reaction buffer, 1.5 mM of MgCl_2_, 0.2 mM of each dNTP, 10 pmol/mL of each primer and 0.5 U/mL of HotStar Taq polymerase (Qiagen, Hilden, Germany). The PCR reaction was set at 94 °C for 3 min, followed by 35 cycles of 94 °C for 40 s, 53 °C for 1.15 min, 72 °C for 1.15 min and 72 °C for 7 min. The gene *prs* was included in the reaction as positive control for *Listeria* sp. *Listeria monocytogenes* F6254 (serotype1/2c) was used as positive control for the genes *lmo0737* and *lmo1118*; *Listeria monocytogenes* F4555 (serotype 4b), as positive control for the genes *ORF2819* and *ORF2110*. The sequence of the primers and probe used in the molecular assays are sown in Table 1.

The PCR products were separated in a 2% agarose gel, stained by ethidium bromide and visualized on a UV transiluminator coupled to a digital image analyzer (Kodak EDAS 290). 

### 2.5. Genotypic Similarity

Pulsed-field gel electrophoresis (PFGE) was used to separate LM isolates according to their genetic similarity. This assay followed the PulseNet/CDC protocol for LM and adopted *ApaI* as the primary restriction enzyme [23]. *Salmonella* Braenderup H9812 was digested by the *XbaI* enzyme and used as a molecular weight standard [24]. The amplified fragments were separated in a 1% agarose gel on a CHEF-DR III (Bio-Rad, Hercules, CA, USA) equipment, stained by ethidium bromide and visualized in a UV transiluminator coupled to a digital image analyzer (Kodak EDAS 290). Results were interpreted using the Gel-J software.

The PFGE profiles were considered indistinguishable when exhibiting 100% similarity. Therefore, a single band difference was sufficient to define two PFGE profiles as different. Taking these characteristics into consideration, genetic similarity between isolates was formally tested using the Dice similarity coefficient with 1.5% tolerance. Additionally, a dendrogram of the PFGE profiles was constructed based on the UPGMA hierarchical clustering method [25].

### 2.6. Antibiotic Resistance

The six LM isolates were tested for antibiotic resistance using the disk-diffusion technique, as described by supplements M02 and M100 of the Clinical and Laboratory Standards Institute [26,27]. A total of 17 antibiotics from 11 different classes were assessed (Table 2). For that, LM isolates were incubated at 35 °C in 5 mL of BHI broth until tubes reached a turbidity equivalent to that of a McFarland standard of 0.5. Aliquots of these solutions were spread above Mueller-Hinton agar, antibiotic disks were dispensed on the agar surface and plates were incubated at 35 °C for 20 h. As no specific criteria on zone diameter breakpoints for LM are available, results were interpreted according to *Staphylococcus* spp. standards. Strains of *Escherichia coli* (ATCC 25922), *Staphylococcus aureus* (ATCC 25923) and *Pseudomonas aeruginosa* (ATCC 27853) were used as internal controls to validate antibiotic disc performance.

For each antibiotic tested, isolates were classified into one of three possible phenotypes: susceptible, intermediate resistance or resistant. Strains that showed resistance to three or more antibiotic classes were considered multi-drug resistant. At all stages, isolates were tested in duplicate.

### 2.7. Disinfectant Susceptibility

The six LM isolates were used to assess the minimal inhibitory concentration (MIC) of five chemical disinfectants by the broth macrodilution technique, as described in the supplements M07 and M100 of the Clinical and Laboratory Standards Institute [27,28]. For that, stock solutions eight times more concentrated than the manufacturers’ recommended concentration were prepared for each disinfectant in sterile BHI broth. Manufacturers recommended concentrations were 187.5 mg/L for peracetic acid, 5000 mg/L for benzalkonium chloride, 20,000 mg/L for chlorhexidine, 2400 mg/L for sodium hypochlorite and 2000 mg/L for quaternary ammonium. From each of the stock solutions, 19 serial two-fold dilutions were prepared. Solutions were then inoculated with suspensions of the isolates (McFarland standard of 0.5), reaching a final concentration of 5 × 10^5^ CFU/mL.

After an incubation period of 20 h at 35 °C, MIC for each disinfectant was considered as the lowest concentration with no bacterial growth (no turbidity). The lack of bacterial growth was confirmed by plating aliquots of these tubes on Mueller-Hinton agar (35 °C for 24 h). At all stages, isolates were tested in duplicate.

## 3. Results

**Figure 1:**
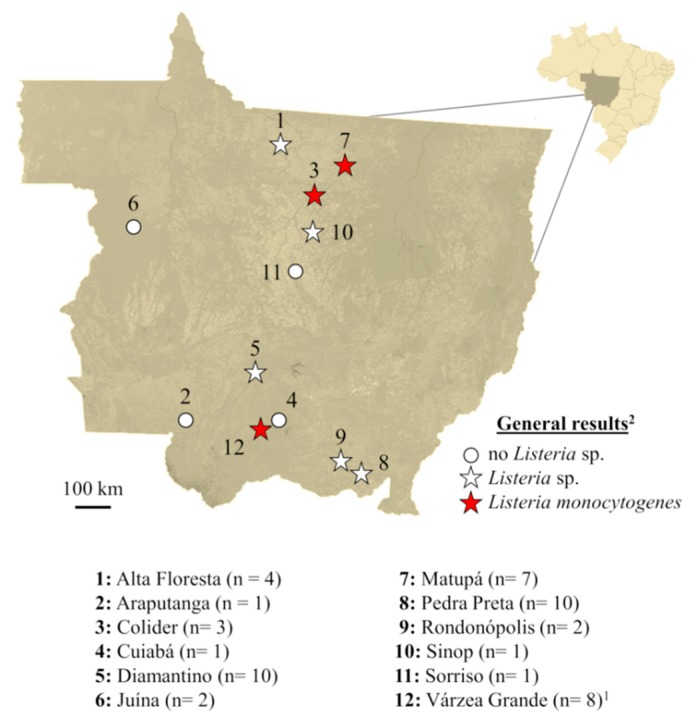
Geographic location (city) of processing plants, number of samples and general results for *Listeria* sp. and *Listeria monocytogenes*. ^1^ In this city, two different processing plants were assessed (*n* = 4/each) and both were positive for *Listeria monocytogenes*. ^2^ Confirmation of *Listeria monocytogenes* were obtained by morphology/biochemistry and real-time PCR.

From the total of 50 samples assessed in this study, 18 (36%) generated typical *Listeria* sp. colonies on Modified Oxford Agar. From those, six were confirmed as LM by both morphological/biochemical and PCR assays, leading to a prevalence of 12% (6/50) of the pathogen. *Listeria* sp. isolates came from beef obtained in nine different processing plants, but LM was confirmed in only four of them (Figure 1).

**Figure 2:**
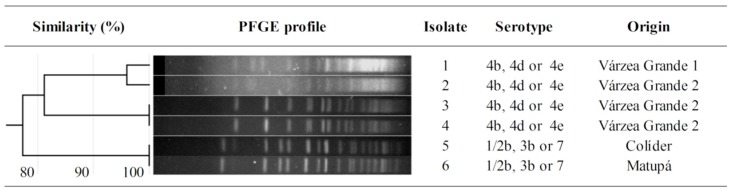
Genotypic similarity and serotyping of *Listeria monocytogenes* isolated from beef obtained in export-authorized processing plants of Mato Grosso, Brazil. Genotypic similarity was formally tested using the Dice similarity coefficient with 1.5% tolerance.

Among the serotype groups evaluated by multiplex PCR, serotype 4 was the most prevalent (Figure 2). This serotype (4b, 4d or 4e) was isolated from samples obtained in the processing plants Várzea Grande 1 and 2, exclusively. Among them, only isolates 3 and 4 (originally from the processing plant Várzea Grande 2) demonstrated 100% homology in the PFGE profile. Serotype group 1/2b, 3b or 7 was isolated from samples obtained in the processing plants Colíder and Matupá. Even though they had different origins, these two isolates (5 and 6) exhibited identical pulsotypes.

Using a broader approach to compare PFGE profiles within LM isolates, we observed that strains originated from processing plants Várzea Grande 1 and 2 had more than 81% similarity. However, by comparing the group of isolates from processing plants Várzea Grande 1 and 2 with that from processing plants Colíder and Matupá, no more than 76% similarity was observed.

In regard to the antimicrobial resistance assays, LM isolates were susceptible to most of the assessed antibiotics, although high rates of resistance were detected to sulfonamides, cefoxitin and cefepime (Table 3). Only the isolate 5 was considered susceptible to sulfonamides.

A similar situation occurred for disinfectants: MICs of benzalkonium chloride, chlorhexidine, peracetic acid and quaternary ammonium were lower than their recommended concentration in all isolates (Table 4). Nevertheless, isolates 1 and 6 showed MIC of 7200 mg/L for sodium hypochlorite, a concentration three times higher than that recommended by the manufacturer.

## 4. Discussion

Our results indicated a 12% prevalence of LM in export-approved beef from Mato Grosso, a number very similar to that of 11.4% obtained by Palma et al. [29] in beef from Brasília, capital of a neighbor state. Numbers from other countries indicate 23% prevalence of LM in beef from Chile [3], and 23.6% in beef from Italy [30]. This is in contrast to the prevalence of LM in beef from Ethiopia, Poland and Turkey, where lower values ranging from 2.5 to 7.2% have been reported [31,32,33].

It is important to note that despite the confirmed 12% prevalence of LM in beef from Mato Grosso, *Listeria* sp. reached a prevalence three times higher than that. Overall, nine of the 13 processing plants indirectly evaluated here were positive for *Listeria* sp. These findings cause concern as they indicate that these positive plants are also suitable for LM growth. According to Sauders and Wiedmann [34], the high DNA homology within the genus *Listeria* makes the ecology of species very similar, meaning they require analogous conditions to grow. This is a strong indication that more intensive measures of cleaning and disinfection must be adopted by the beef processing industry of Mato Grosso. This situation puts at risk not only the Brazilian customer, but also the population of the countries that import beef from those processors.

Most of the LM isolates belonged to serotype 4 (4b, 4d or 4e), but serotype group 1/2b, 3b and/or 7 were also detected. Unfortunately, the multiplex PCR protocol adopted here does not allow the complete differentiation of serotypes within these groups. Although serotyping provides biological context for the intra phylogenetic and phenotypic relationships between strains [35], the separation of LM into lineages allows a broader interpretation of the evolutionary aspects related to the origin of strains and provides better foundation to analyze the differences on their virulence and antimicrobial resistance. Genetic similarities between serotypes grant their classification into four different lineages [36]: lineage I is represented by serotypes 1/2b, 3b, 3c and 4b; lineage II, by serotypes 1/2a, 1/2c and 3a; lineages III and IV, by serotypes 4a, 4b and 4c.

From this perspective, LM isolated from plants Colíder and Matupá can be exclusively classified as lineage I, but LM isolated from plants Várzea Grande 1 and 2 could be part of lineages I, III or IV. Lineages I and II have been identified in the great majority of the clinical cases of listeriosis in humans [37,38], especially the serotype 1/2a (lineage II) which is also the most prevalent in food [39,40]. In a previous study by our research group, LM 1/2a was the predominant serotype (94.6%) isolated from samples of chicken meat and chicken meat processing environment in Mato Grosso [41]. In the present study, no serotype 1/2a was identified in beef from Mato Grosso.

Despite the greater occurrence of LM lineage II in clinical cases of human listeriosis, lineage I is usually more virulent due to a premature stop codon frequently present in the virulence gene *inlA* of LM lineage II [42,43], which disrupts the translation of the gene and reduces bacteria potential for cellular invasiveness. In fact, by analyzing 38 LM isolates from clinical (human) and non-clinical (food and food-related environment) samples obtained in Chile, Toledo et al. [44] found that the majority of the clinical isolates belonged to lineage I. Considering this, LM isolated here has a great potential of being harmful to humans, especially those from processing plants Colíder and Matupá, which are unambiguously assigned to lineage I.

The distance between processing plants proved to be an important element on the genetic similarity between isolates. Clone strains were detected in the processing plant Várzea Grande 2 (isolates 3 and 4) and also in the processing plants Colíder and Matupá (isolates 5 and 6). Even though these last processors were located in different cities, the distance between them was short (approximately 120 km). In this condition, it is plausible to consider that animals raised in the same farm were slaughtered in both plants; or even that the personnel and trucks used to transport the animals were the same. These shared items, along with failures in the cleaning and disinfection procedures adopted by the industries, might explain the finding of clonal strains in samples from different plants. In contrast, less than 76% genetic similarity was detected when comparing the group of LM isolated from plants Várzea Grande 1 and 2 with that isolated from plants Colíder and Matupá. The distance between these sites ranged from 650 to 690 km.

With respect to the antimicrobial susceptibility assays, results revealed low levels of antibiotic resistance. The exceptions were: i) cefoxitin and cefepime—not surprising as LM is naturally resistant to subclasses of cephems, especially cephalosporin [45]; ii) sulfonamides—this phenotype is of importance as the combination of trimethoprim and sulfonamides is the treatment of choice against listeriosis in penicillin-allergic patients [46]. However, resistance to the combination of trimethoprim and sulfamethoxazole was null in the present study.

Multi-drug resistant LM were previously isolated from beef in Italy [30] and Poland [32]. Resistance included drugs traditionally used to treat listeriosis, such as ampicillin, and others including vancomycin, tetracycline, erythromycin and oxacillin. Our results may indicate that the exposure of Mato Grosso’s cattle to some of these molecules is low.

As well as that observed for antibiotics, resistance to disinfectants was sporadic in this study. All the isolates presented MICs below the recommended concentrations of benzalkonium chloride, chlorhexidine, peracetic acid and quaternary ammonium. This is in agreement with the results from Stoller et al. [47], who described no resistance to the typical usage concentration of benzalkonium chloride and peracetic acid in LM strains isolated in a Swiss meat-processing facility over a four-year period. However, two isolates (1 and 6) exhibited MIC of sodium hypochlorite three times higher than its recommended concentration. Resistance to sodium hypochlorite was already described in LM isolated from dairy facilities in Brazil [48]. The high availability and affordable price of this molecule make it one of the most used disinfectants by the food industry and production farms.

The presence of resistant strains in this study evinces not only the high usage of sodium hypochlorite in the production chain, but could also imply that the procedures of cleaning and disinfection are not being efficient enough to avoid the emergence and persistence of resistant strains in the processing environment. *Listeria* sp. are microorganisms of particular interest in this topic because their great ability to produce biofilms and protect themselves from the action of disinfectants [16,49].

Differences in the choice of disinfectants, concentration of usage and efficacy of the cleaning and disinfection procedures between plants can also be the reason for the discrepant resistance profiles observed in isolates with 100% genetic homology. This situation occurred for isolates 5 and 6, clones from different plants that showed, respectively, the lowest and the highest MIC for sodium hypochlorite. Same reasoning can be applied to antibiotic resistance: isolate 5 was susceptible to sulfonamides, while isolate 6 was not. These phenotypic differences between clone strains reinforce the rule of selective pressure as a determinant for the expression of antimicrobial resistance, even in microorganisms that share the same genotype.

## 5. Conclusions

The estimated prevalence of *Listeria monocytogenes* in export-approved beef from Mato Grosso is 12%, but the prevalence of *Listeria* sp. reaches a value three times higher than that. Several serotypes and genotypic profiles circulate in the state; however, apparently clonal strains could be isolated in samples from different processing plants close to each other.

Antibiotic resistance was not an issue within the isolates, especially considering the molecules frequently used to treat listeriosis in humans. However, high level of resistance to the disinfectant sodium hypochlorite was detected in different strains. Overall, our results suggest that more intensive measures of cleaning and disinfection should be adopted by the beef industry of Mato Grosso.

## Figures and Tables

**Table 1:** Sequence and serotype specificity of primers and probes used in this study.

Code	Sequence (5′–3′)	Specificity
*hlyA*	F: AGAAGTNATTAGTTTTAAACAAATTTACTATAACG	*Listeria monocytogenes*
	R: AACTGCTCTTTAGTNACAGCTTTGC	
*hlyA Probe*	FAM –TGAACCTACANGACCTTCC– MGB	*Listeria monocytogenes*
*prs*	F: GCTGAAGAGATTGCGAAAGAAG	*Listeria sp.*
	R: CAAAGAAACCTTGGATTTGCGG	
*lmo0737*	F: AGGGCTTCAAGGACTTACCC	*Listeria monocytogenes* 1/2a, 1/2c, 3a, 3c
	R: ACGATTTCTGCTTGCCATTC	
*lmo1118*	F: AGGGGTCTTAAATCCTGGAA	*Listeria monocytogenes* 1/2c, 3c
	R: CGGCTTGTTCGGCATACTTA	
*ORF2819*	F: AGCAAAATGCCAAAACTCGT	*Listeria monocytogenes* 1/2b, 3b, 4b, 4d, 4e
	R: CATCACTAAAGCCTCCCATTG	
*ORF2110*	F: AGTGGACAATTGATTGGTGAA	*Listeria monocytogenes* 4b, 4d, 4e
	R: CATCCATCCCTTACTTTGGAC	

Adapted from Doumith et al. [22].

**Table 2:** Antibiotics and zone diameter breakpoints used in the disk-diffusion assay.

Antibiotic	Code	Class	Disk Content (µg)	Zone Diameter Breakpoints (mm) for Each Phenotype		
				S	I	R
Ciprofloxacin	CIP	Fluoroquinolone	5	≥21	16–20	≤15
Enrofloxacin	ENR	Fluoroquinolone	5	≥18	15–17	≤14
Sulfonamides	SSS	Folate pathway inhibitor	300	≥17	13–16	≤12
Trimethoprim	TMP	Folate pathway inhibitor	5	≥16	11–15	≤10
Trimethoprim + sulfamethoxazole	SXT	Folate pathway inhibitor	23.75	≥16	11–15	≤10
Ampicillin	AMP	Penicillin	10	≥29	-	≤28
Nitrofurantoin	NIT	Nitrofuran	300	≥17	15–16	≤14
Gentamicin	GEN	Aminoglycoside	10	≥15	13–14	≤12
Rifampin	RIF	Ansamycin	5	≥20	17–19	≤16
Chloramphenicol	CHL	Phenicol	30	≥18	13–17	≤12
Florfenicol	FLF	Phenicol	30	≥18	13–17	≤12
Erythromycin	ERY	Macrolide	15	≥23	14–22	≤13
Azithromycin	AZI	Macrolide	15	≥18	14–17	≤13
Imipenem	IPM	Carbapenem	10	≥22	-	≤21
Tetracycline	TET	Tetracycline	30	≥19	15–18	≤14
Cefoxitin	FOX	Cephem	30	≥22	-	≤21
Cefepime	FEP	Cephem	30	≥24	21–23	≤20

S = susceptible; I = intermediate; R = resistant.

**Table 3:** Antibiotic resistance profile of *Listeria monocytogenes* isolated from beef obtained in export-authorized processing plants of Mato Grosso, Brazil.

Antibiotic	Code	Resistance Profile ^1^					
		Isolate 1	Isolate 2	Isolate 3	Isolate 4	Isolate 5	Isolate 6
Ciprofloxacin	CIP	S	S	S	S	S	S
Enrofloxacin	ENR	S	S	S	S	S	S
Sulfonamides	SSS	I	I	R	R	S	R
Trimethoprim	TMP	S	S	S	S	S	S
Trimethoprim + sulfamethoxazole	SXT	S	S	S	S	S	S
Ampicillin	AMP	S	S	S	S	S	S
Nitrofurantoin	NIT	S	S	S	S	S	S
Gentamicin	GEN	S	S	S	S	S	S
Rifampin	RIF	S	S	S	S	S	S
Chloramphenicol	CHL	S	S	S	S	S	S
Florfenicol	FLF	S	S	S	S	S	S
Erythromycin	ERY	S	S	S	S	S	S
Azithromycin	AZI	S	S	S	S	S	S
Imipenem	IPM	S	S	S	S	S	S
Tetracycline	TET	S	S	S	S	S	S
Cefoxitin	FOX	R	R	R	R	R	R
Cefepime	FEP	R	S	R	R	R	R

S = susceptible; I = intermediate; R = resistant. ^1^ Determined by disk diffusion following the recommendations of the Clinical and Laboratory Standards Institute [26,27]. Isolate 1 was obtained from beef processed at plant Várzea Grande 1; isolates 2, 3 and 4 at plant Várzea Grande 2; isolate 5 at plant Colíder; isolate 6 at plant Matupá.

**Table 4:** Minimal inhibitory concentration of chemical disinfectants for *Listeria monocytogenes* isolated from beef obtained in export-authorized processing plants of Mato Grosso, Brazil.

Chemical Disinfectant ^2^	Recommended Concentration (mg/L)	Minimal Inhibitory Concentration (mg/L) ^1^						
		Isolate 1	Isolate 2	Isolate 3	Isolate 4	Isolate 5	Isolate 6	Average
Benzalkonium chloride	5000	0.6	9.8	4.9	4.9	4.9	1.2	4.4
Chlorhexidine	20,000	0.2	0.2	0.4	0.2	1.6	0.4	0.5
Peracetic acid	187.5	2.9	23.4	11.7	46.9	23.4	1.5	18.3
Quaternary ammonium	2000	2.0	31.3	7.8	31.3	15.6	3.9	15.3
Sodium hypochlorite	2400	7200	1800	1800	1800	450	7200	3375

^1^ Determined by broth macrodilution, as described in the supplements M07 and M100 of the Clinical and Laboratory Standards Institute [27,28]. ^2^ Benzalkonium chloride (CMT, Várzea Grande, Brazil), chlorhexidine (Rioquimica, São José do Rio Preto, Brazil), peracetic acid (Mustang Pluron, Brazil), quaternary ammonium (Mustang Pluron, Catanduva, Brazil) and sodium hypochlorite (Lima & Pergher, Rio de Janeiro, Brazil) were tested in 20 concentrations, comprising stock solutions eight times more concentrated than the recommended dosage and 19 serial two-fold dilutions. Isolate 1 was obtained from beef processed at plant Várzea Grande 1; isolates 2, 3 and 4 at plant Várzea Grande 2; isolate 5 at plant Colíder; isolate 6 at plant Matupá.
